# Supplementary material for: DNA-PKcs Inhibition Sensitizes Human Chondrosarcoma Cells to Carbon Ion Irradiation via Cell Cycle Arrest and Telomere Capping Disruption
Source: Int J Mol Sci. 2024 Jun 4;25(11):6179. doi: 10.3390/ijms25116179 (PMC11173223; doi:10.3390/ijms25116179)
Supplement: Supplementary file 1 [file ijms-25-06179-s001.zip › Suppl Table S1_qPCR values.pdf]

		**X-ray**				**C-ions**			
**target**	**cell line**	**ctrl 0 Gy**	**AZD 0 Gy**	**ctrl 8 Gy**	**AZD 8 Gy**	**ctrl 0 Gy**	**AZD 0 Gy**	**ctrl 8 Gy**	**AZD 8 Gy**
**c-Myc**	SW-1353	1.00±0.1	1.10±0.2<br>*	1.01±0.11	1.38±0.4<br>***<br>#	1.00±0.0	1.35±0.3<br>**	1.82±0.3<br>***	2.14±0.6<br>***<br>##
	Cal78	1.05±0.1	1.33±0.2<br>***	1.09±0.2	1.47±0.3<br>***	1.00±0.0	1.27±0.4<br>**	1.55±0.4<br>***	1.81±0.5<br>***<br>##
**cyclin D1**	SW-1353	1.00±0.0	1.15±0.2<br>*	2.29±0.6<br>***	2.76±0.8<br>***<br>###	1.00±0.0	0.93±0.3	1.67±0.4<br>***	1.93±0.7<br>***<br>###
	Cal78	1.00±0.0	1.53±0.3<br>***	2.42±0.7<br>***	2.89±0.7<br>***<br>###	1.00±0.0	1.12±0.1	1.37±0.4<br>**	1.34±0.5<br>*
**survivin**	SW-1353	1.00±0.0	0.97±0.2	0.57±0.1<br>***	0.42±0.2<br>***<br>###	1.00±0.0	0.79±0.1<br>***	0.38±0.2<br>***	0.23±0.1<br>***<br>###
	Cal78	1.00±0.0	1.03±0.1	0.96±0.2	0.87±0.2<br>*<br>#	1.00±0.1	0.83±0.3	0.45±0.3<br>***	0.42±0.1<br>***<br>###
**cyclin B**	SW-1353	1.00±0.0	0.83±0.1<br>***	0.59±0.1<br>***	0.34±0.1<br>***<br>###	1.00±0.0	1.16±0.1	0.64±0.2<br>***	0.53±0.2<br>***<br>###
	Cal78	1.01±0.0	0.99±0.1	0.79±0.1<br>***	0.51±0.1<br>***<br>###	1.00±0.0	0.81±0.2<br>***	0.62±0.1<br>***	0.59±0.1<br>***<br>###
**CDK1**	SW-1353	1.01±0.0	0.83±0.1<br>***	0.97±0.1	0.67±0.1<br>***<br>##	1.01±0.0	1.50±0.3<br>***	0.52±0.1<br>***	0.59±0.2<br>***<br>###

	Cal78	1.00±0.0	0.98±0.1	1.53±0.2<br>***	1.23±0.2<br>**<br>##	1.00±0.0	1.20±0.3<br>*	1.19±0.2<br>**	1.18±0.2<br>**
**WEE1**	SW-1353	1.00±0.0	0.79±0.1<br>***	0.60±0.1<br>***	0.49±0.1<br>***<br>###	1.00±0.1	1.26±0.3<br>**	0.91±0.1<br>*	0.56±0.2<br>***<br>###
	Cal78	1.00±0.0	0.93±0.1<br>*	0.89±0.1<br>***	0.74±0.1<br>***<br>###	1.00±0.1	1.01±0.1	0.81±0.1<br>***	0.86±0.2<br>*<br>#
**HSP27**	SW-1353	1.00±0.0	1.18±0.2<br>***	0.94±0.2	0.75±0.2<br>***<br>###	1.00±0.0	1.39±0.6	0.84±0.3	0.88±0.2<br>*<br>#
	Cal78	1.00±0.0	0.93±0.1<br>**	0.75±0.2<br>***	0.59±0.2<br>***<br>###	1.00±0.0	0.75±0.2<br>***	0.60±0.2<br>***	0.54±0.2<br>***<br>##
**AMPK**	SW-1353	1.00±0.0	1.14±0.2<br>**	1.43±0.3<br>***	1.75±.04<br>***<br>###	1.00±0.1	1.14±0.2<br>*	1.31±0.4<br>**	2.22±0.4<br>***<br>###
	Cal78	1.00±0.0	1.60±0.5<br>**	1.16±0.3	1.67±0.4<br>**	1.00±0.0	1.04±0.3	1.01±0.3	1.27±0.3<br>**<br>#
**XRCC4**	SW-1353	1.00±0.0	0.87±0.1<br>***	1.56±0.3<br>***	1.59±0.3<br>***<br>###	1.00±0.0	1.14±0.3	1.70±0.2<br>***	0.85±0.1<br>***<br>##
	Cal78	1.00±0.0	1.01±0.2	1.40±0.1<br>***	1.51±0.2<br>***<br>###	1.00±0.0	0.69±0.1<br>***	1.78±0.4<br>***	1.02±0.1<br>#
**MDM2**	SW-1353	1.00±0.1	0.67±0.1<br>***	2.73±0.9<br>***	3.76±1.1<br>***<br>###	1.00±0.1	1.17±0.4	3.24±1.0<br>***	4.55±1.8<br>***<br>###

	Cal78	1.00±0.1	1.07±0.3	0.74±0.1<br>***	1.58±0.4<br>***<br>###	1.00±0.1	0.77±0.2<br>*	1.55±0.3<br>***	1.37±0.4<br>***<br>###
**p53**	SW-1353	1.00±0.0	0.99±0.3	1.30±0.3<br>**	1.06±0.3	1.00±0.0	1.37±0.3<br>***	1.93±0.4<br>***	1.66±0.6<br>***<br>###
	Cal78	1.00±0.0	1.16±0.3	1.81±0.3<br>***	1.64±0.3<br>***<br>###	1.00±0.0	0.90±0.2	1.62±0.5<br>***	1.42±0.2<br>***<br>###

Table S1. Relative gene expression analysis after irradiation (IR) of 8 Gy X-ray, respectively 8 Gy C-ions and the combined treatment with the DNA-PKcs inhibitor AZD7648 (mean ± SD; *n* = 6; measured in triplicates). Statistical significances to the untreated controls (ctrl 0 Gy) are defined as follows: * $p < 0.05$; ** $p < 0.01$; *** $p < 0.001$. Statistical significances between the 3 μM AZD7648 group and the combined treatment with IR are presented as # $p < 0.05$; ## $p < 0.01$; ### $p < 0.001$.
