# Supplementary material for: DNA-PKcs Inhibition Sensitizes Human Chondrosarcoma Cells to Carbon Ion Irradiation via Cell Cycle Arrest and Telomere Capping Disruption
Source: Int J Mol Sci. 2024 Jun 4;25(11):6179. doi: 10.3390/ijms25116179 (PMC11173223; doi:10.3390/ijms25116179)
Supplement: Supplementary file 1 [file ijms-25-06179-s001.zip › Suppl Table S2_protein band quantification.pdf]

		X-ray				C-ions			
		Δ ratio to ctrl 0 Gy				Δ ratio to ctrl 0 Gy			
**target**	**cell line**	**ctrl 8 Gy**	**1 μM AZD 8 Gy**	**3 μM AZD 8 Gy**	**10 μM AZD 8 Gy**	**ctrl 8 Gy**	**1 μM AZD 8 Gy**	**3 μM AZD 8 Gy**	**10 μM AZD 8 Gy**
**cyclin B (24 h)**	SW-1353	1.77±0.5	0.71±0.2	0.79±0.11	0.36±0.0 **	3.71±1.7 *	2.02±1.2	1.22±0.2	0.84±0.2
	Cal78	1.25±0.6	1.15±0.5	0.42±0.2 *	0.15±0.1 **	2.16±0.8	1.72±1.0	1.44±1.1	0.57±0.2 *
**CDK1 (24 h)**	SW-1353	1.53±0.4	0.96±0.6	0.91±0.8	0.33±0.2 *	2.76±0.7 *	0.85±0.3	0.94±0.2	0.76±0.3
	Cal78	4.2±1.8	2.45±0.9	2.26±0.4 *	1.24±0.2	1.05±0.7	0.79±0.5	1.03±0.6	0.80±0.4
**p53 (24 h)**	SW-1353	2.12±0.6	2.35±0.6	1.98±1.0	0.95±0.2	2.46±1.5 *	2.26±0.4 *	2.90±0.8 *	1.97±0.2 *
	Cal78	0.94±0.1	1.41±0.1	1.53±0.3	0.85±0.5	1.38±0.6	1.46±0.5	1.80±0.5	1.00±0.1
**pAKT (1 h)**	SW-1353	1.75±0.2 *	0.81±0.2	0.37±0.1 **	0.14±0.1 **	1.84±0.3	1.49±0.3	0.82±0.0	0.34±0.3
	Cal78	1.22±0.5	0.50±0.1 *	0.37±0.1 *	0.14±0.0 ***	9.01±1.8 *	4.82±0.2 *	2.83±0.7 *	1.26±0.3

**p-Chk2 (1 h)**	SW-1353	1.82±0.9	3.35±0.5<br>*	11.70±2.5<br>*	14.72±3.4<br>*	3.71±0.2<br>*	5.53±1.2<br>*	8.71±0.4<br>*	15.16±1.0<br>**
	Cal78	1.81±0.9	8.05±1.8<br>*	9.59±2.2<br>*	7.00±1.4<br>*	1.37±0.7	3.89±2.3	7.60±4.0	8.77±4.4
**p-DNA-PKcs (1 h)**	SW-1353	8.86±1.6<br>*	0.96±0.4	0.74±0.4	0.54±0.4<br>**	5.93±2.8	0.97±0.5	0.53±0.1	0.36±0.3
	Cal78	14.69±5.0<br>*	3.55±1.8	2.54±1.8	1.59±0.5	7.73±3.6	1.55±0.6	1.29±0.1	1.36±0.2
**Ku70 (24 h)**	SW-1353	0.85±0.3	0.64±0.1<br>*	0.52±0.1<br>**	0.79±0.2	0.56±0.2	0.38±0.2<br>*	0.51±0.1<br>*	0.46±0.2<br>*
	Cal78	0.79±0.2	0.78±0.2	0.82±0.4	0.67±0.3	0.62±0.2	0.71±0.2	0.79±0.2	0.89±0.2
**Ku80 (24 h)**	SW-1353	0.93±0.3	0.92±0.1	0.85±0.2	0.76±.0.1	0.64±0.1<br>*	0.60±0.2	0.85±0.3	0.63±0.4
	Cal78	0.68±0.2	0.63±0.3	0.67±0.3	0.81±0.1	1.16±0.2	1.13±0.1	1.26±0.3	1.52±0.3
**Artemis (24 h)**	SW-1353	0.57±0.1<br>*	0.48±0.1<br>**	0.53±0.1<br>**	0.34±0.2<br>*	0.65±0.3	0.30±0.1<br>**	0.25±0.1<br>*	0.50±0.1<br>*
	Cal78	0.73±0.3	0.84±0.4	0.51±0.2<br>*	0.44±0.2<br>*	0.81±0.2	0.57±0.2	0.64±0.1<br>**	0.32±0.2<br>*

**DNA-Ligase IV (24 h)**	SW-1353	1.57±0.5	0.71±0.1<br>*	0.70±0.1	0.77±0.2	0.83±0.4<br>*	0.62±0.5	0.86±0.5	0.81±0.5
	Cal78	0.95±0.1	1.07±0.1	1.15±0.4	0.70±0.3	0.98±0.5	0.79±0.5	0.80±0.3	0.65±0.5
**γH2AX (24 h)**	SW-1353	1.98±0.7	4.22±0.8<br>*	4.97±1.2<br>*	3.86±0.4<br>**	2.34±0.1<br>*	4.64±0.2<br>**	5.29±2.3<br>*	3.68±1.5<br>*
	Cal78	2.77±1.5	4.52±2.2	3.37±0.9<br>*	2.00±0.7	2.19±0.8	2.52±2.3	2.85±2.2	2.74±1.7

Table S2. Western blot analysis were performed after irradiation of 8 Gy X-ray, respectively 8 Gy C-ions and the combined treatment with the DNA-PKcs inhibitor AZD7648. Δ ratio values are given in mean± SD (n = 3). Statistical significances to the untreated controls (ctrl 0 Gy) are defined as follows: * $p < 0.05$; ** $p < 0.01$; *** $p < 0.001$. Densiometry of the protein bands was performed using Image J software (https://imagej.net).
