# Supplementary material for: DNA-PKcs Inhibition Sensitizes Human Chondrosarcoma Cells to Carbon Ion Irradiation via Cell Cycle Arrest and Telomere Capping Disruption
Source: Int J Mol Sci. 2024 Jun 4;25(11):6179. doi: 10.3390/ijms25116179 (PMC11173223; doi:10.3390/ijms25116179)

supplementary data

uncropped files

Figures 3, 4 and 6

uncropped files_Figure 3A (SW-1353)

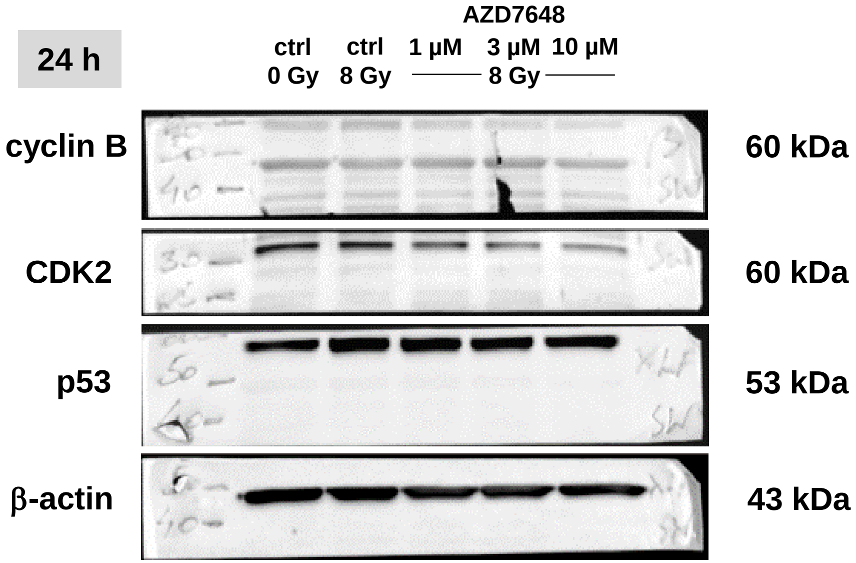

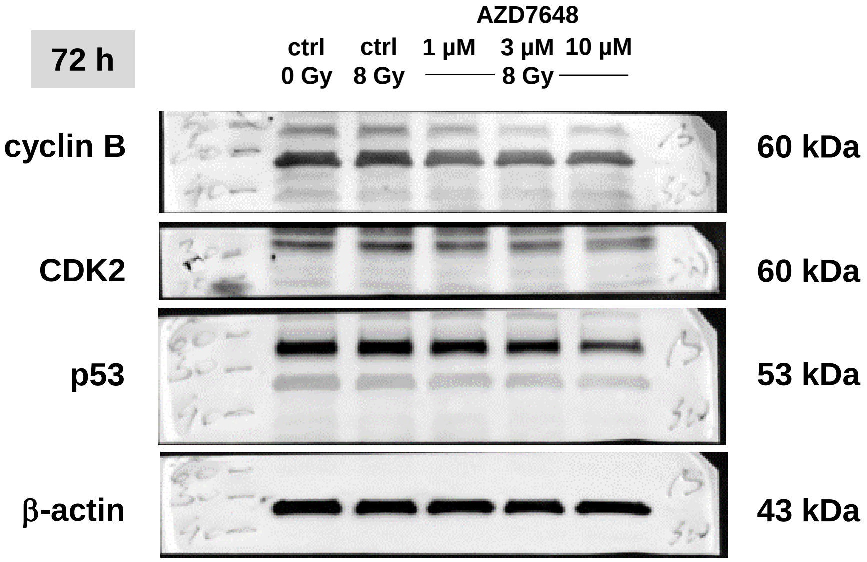

uncropped files_Figure 3A (Cal78)

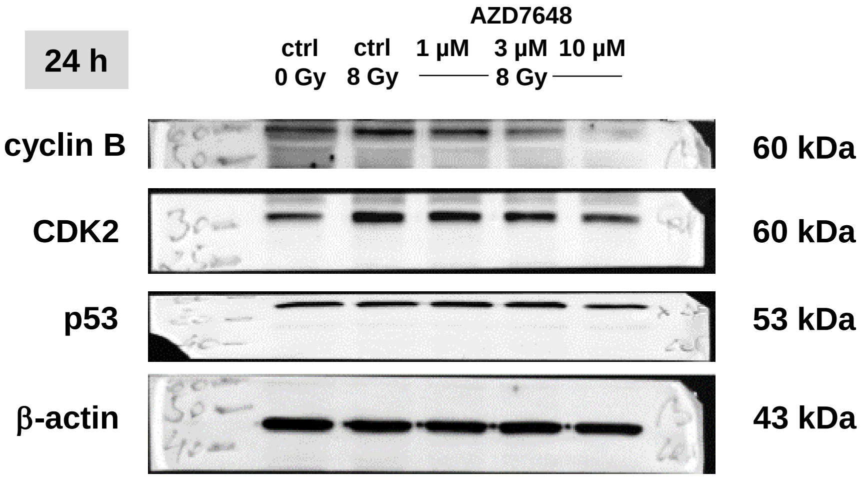

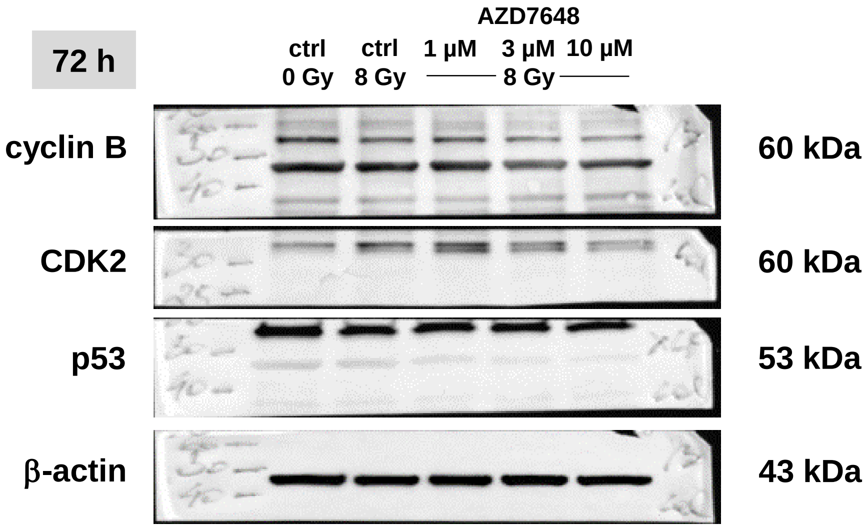

uncropped files_Figure 3B (SW-1353)

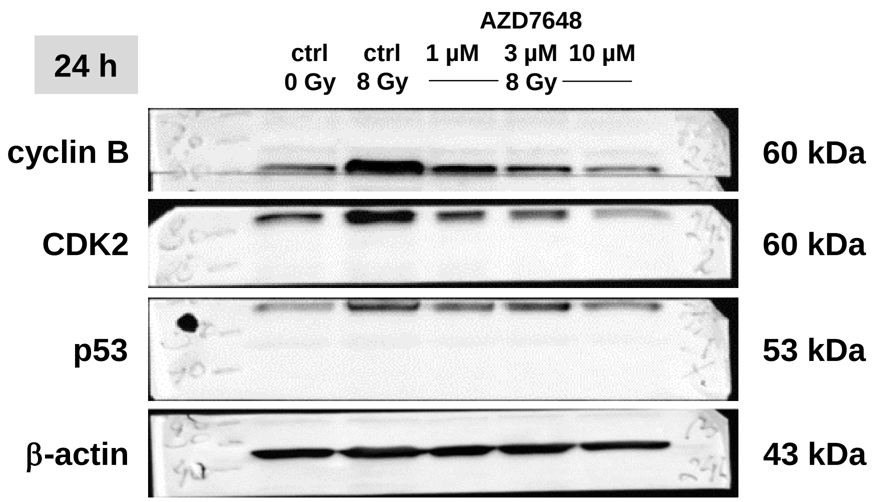

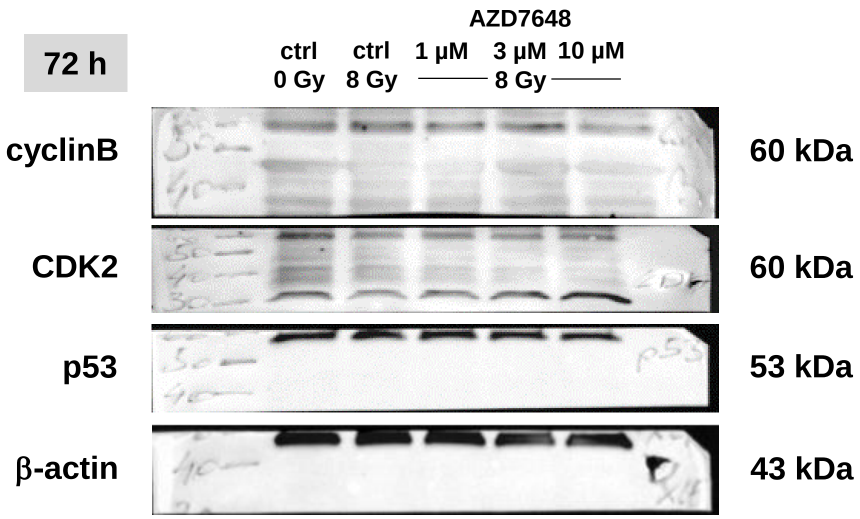

uncropped files_Figure 3B (Cal78)

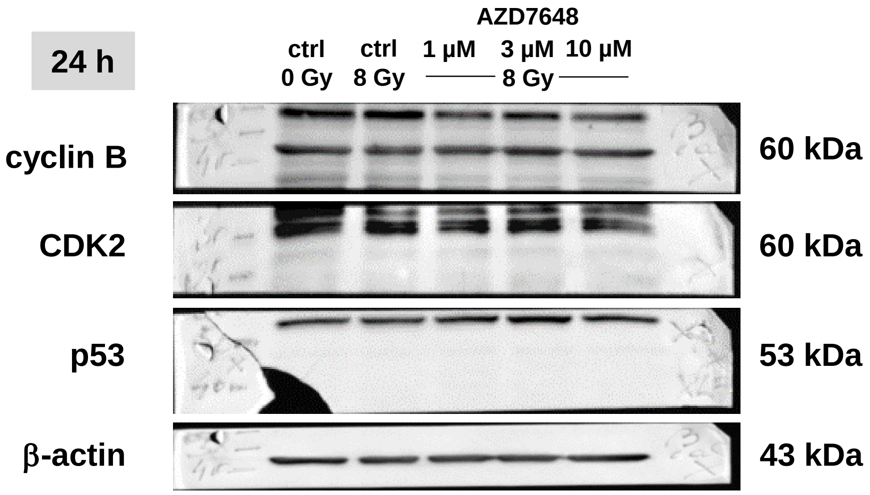

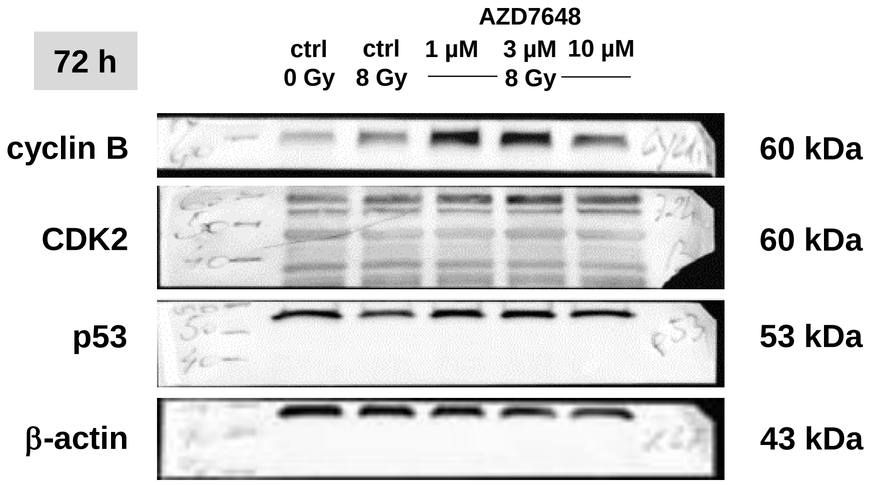

uncropped files_Figure 4A (X-ray)

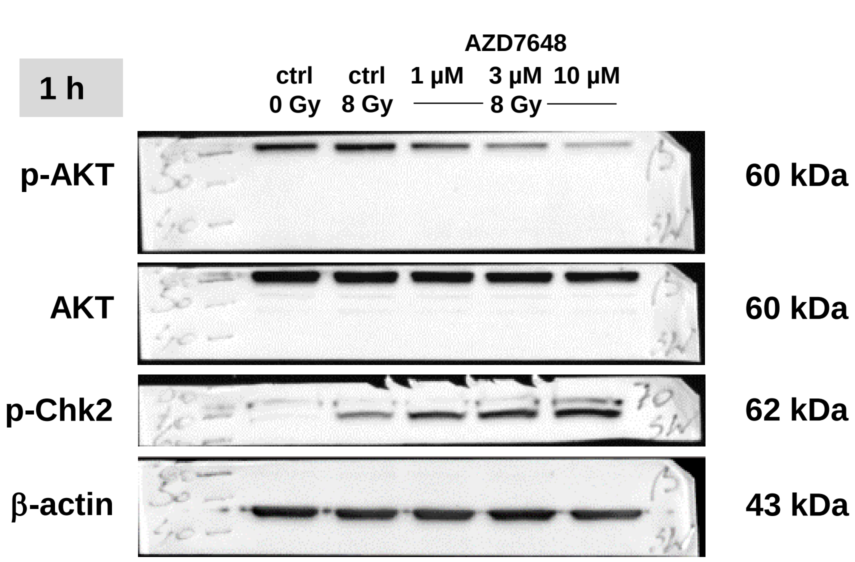

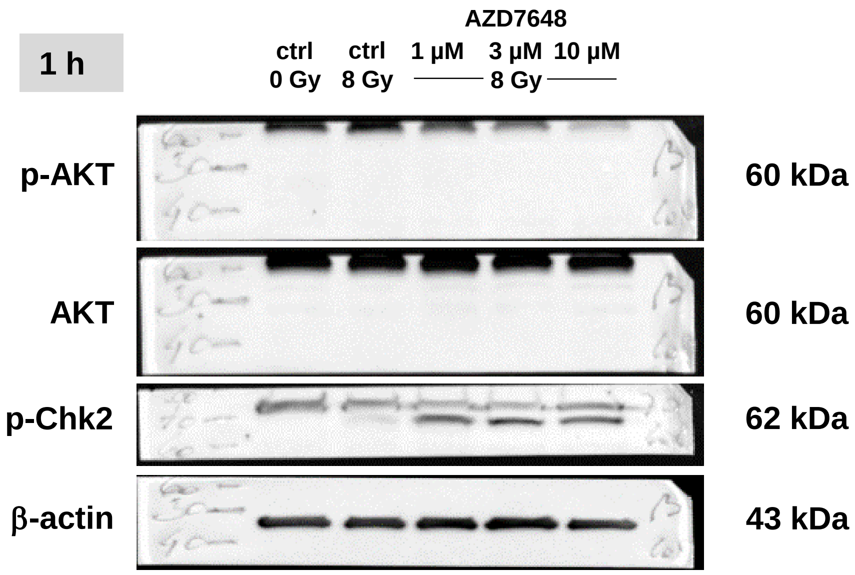

uncropped files_Figure 4B (C-ions)

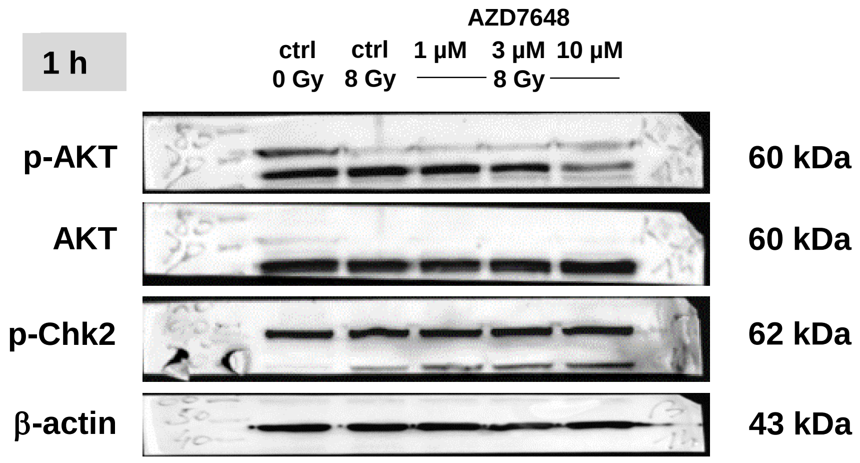

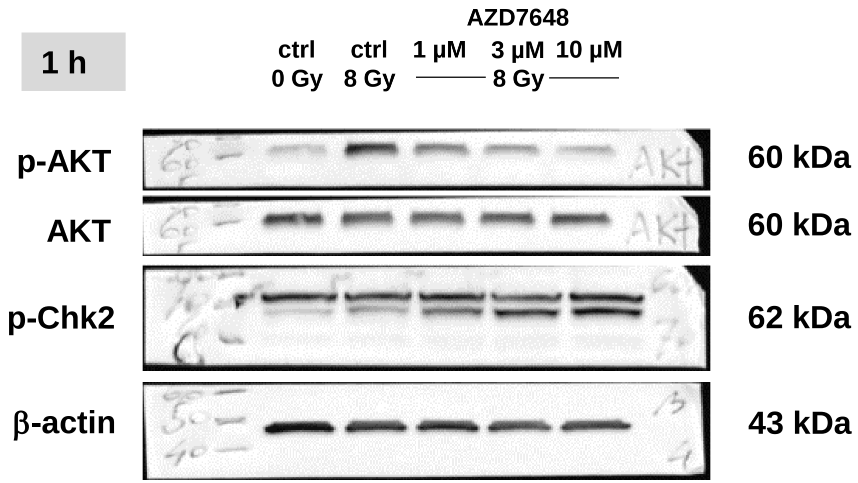

uncropped files_Figure 6A (SW-1353; X-ray)

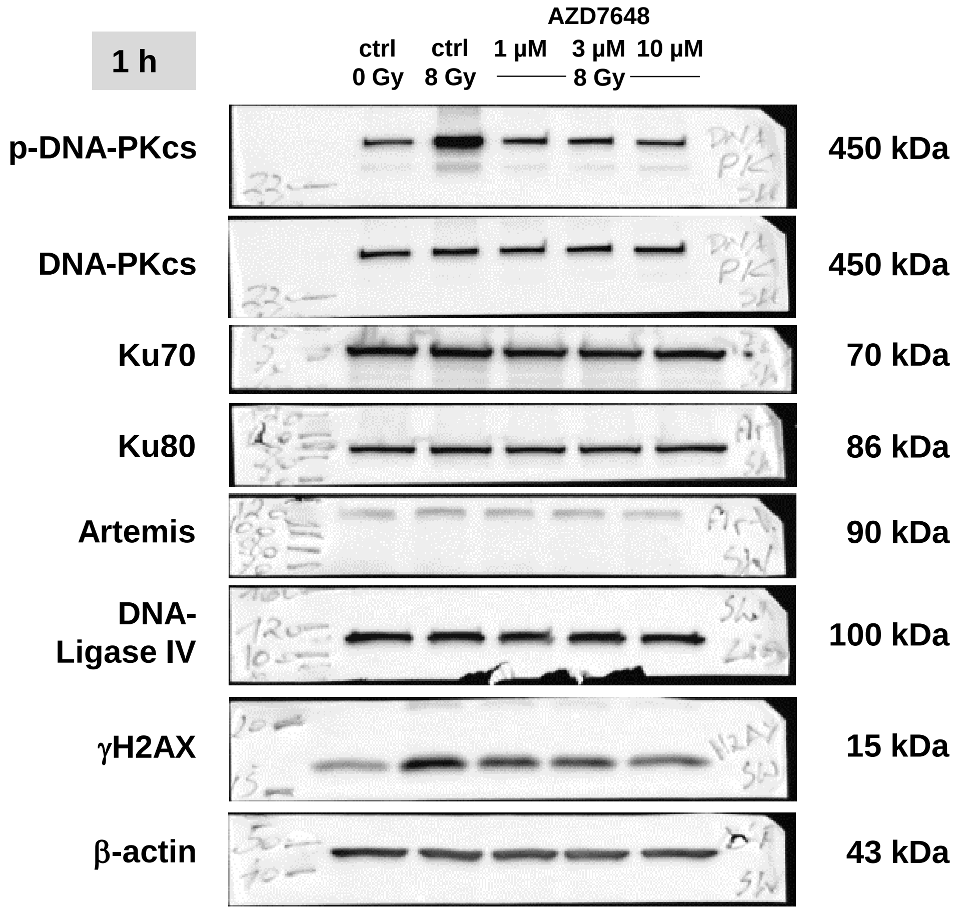

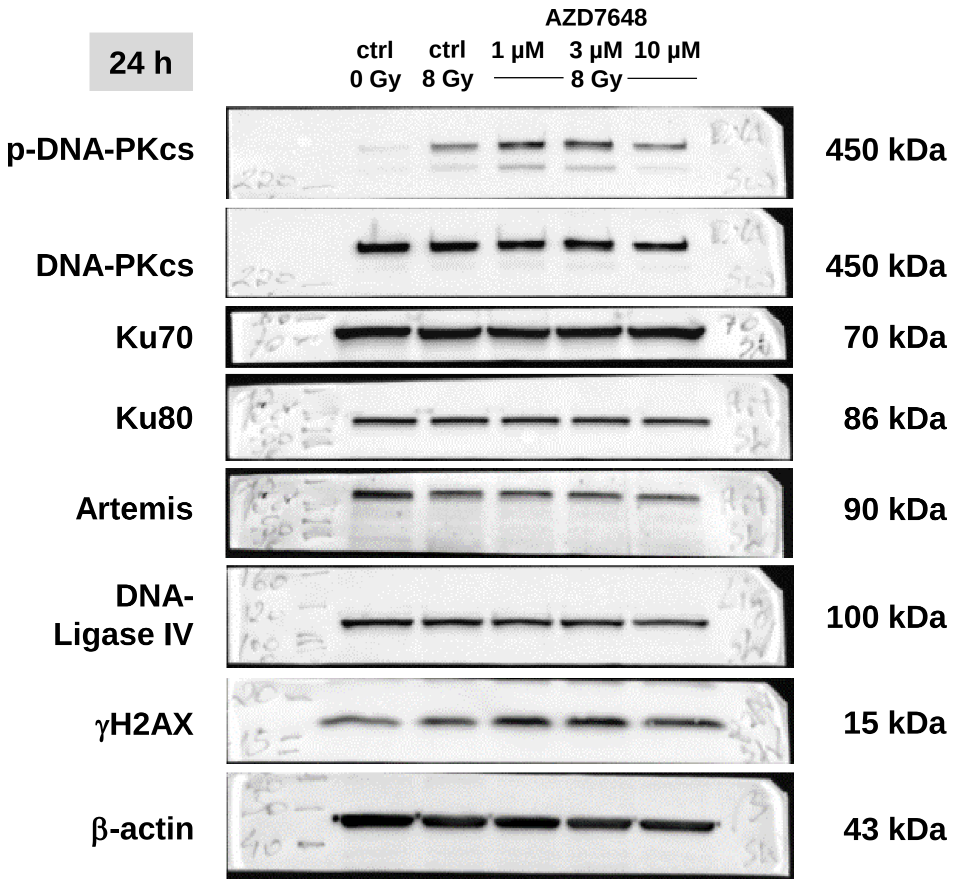

uncropped files_Figure 6A (Cal78; X-ray)

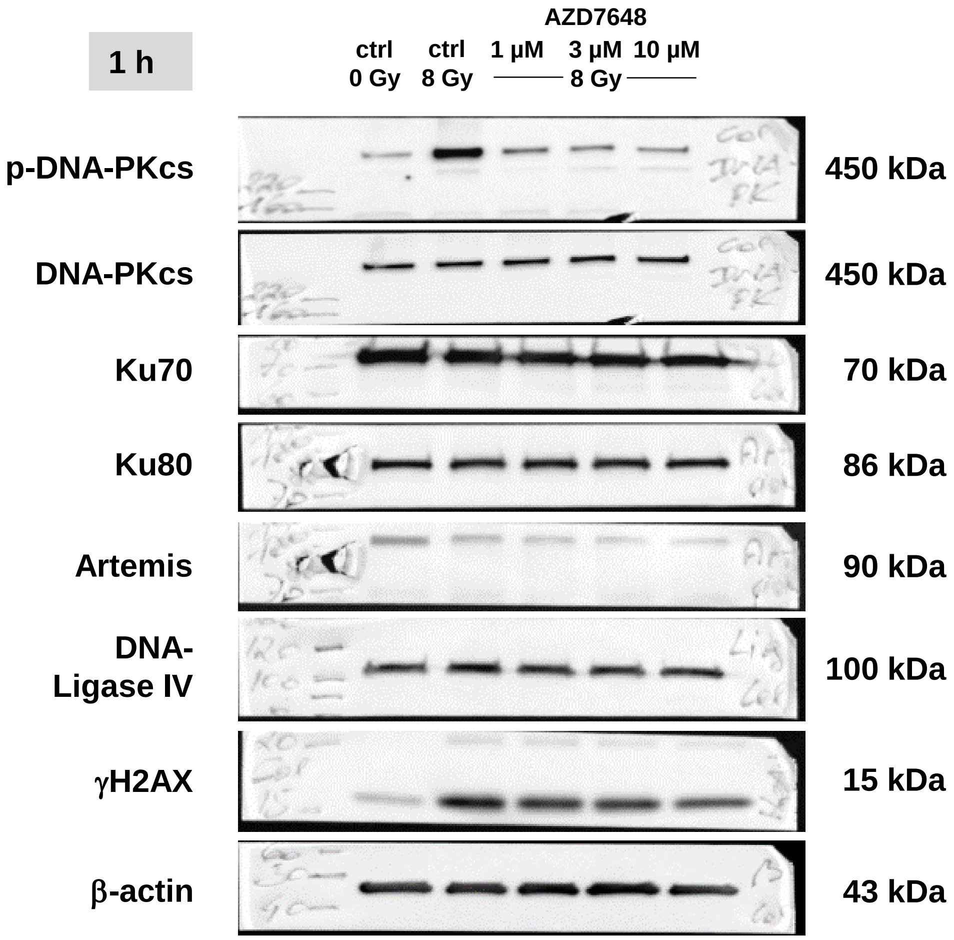

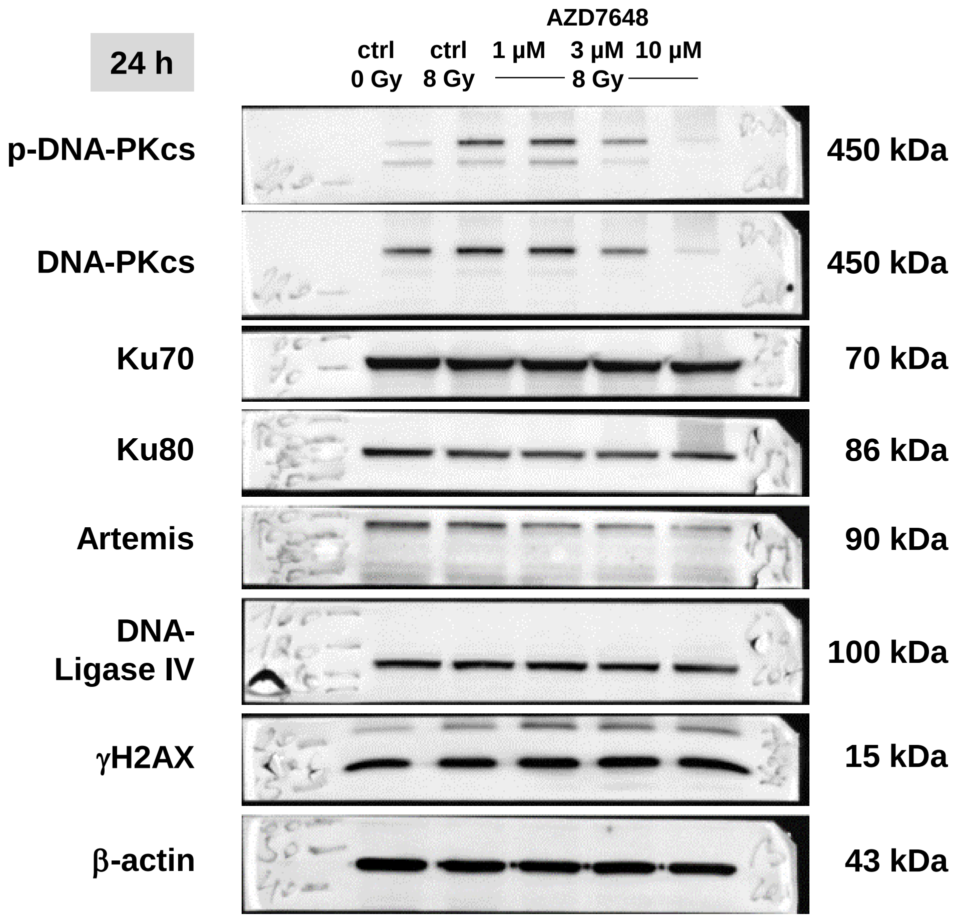

uncropped files_Figure 6B (SW-1353; C-ions)

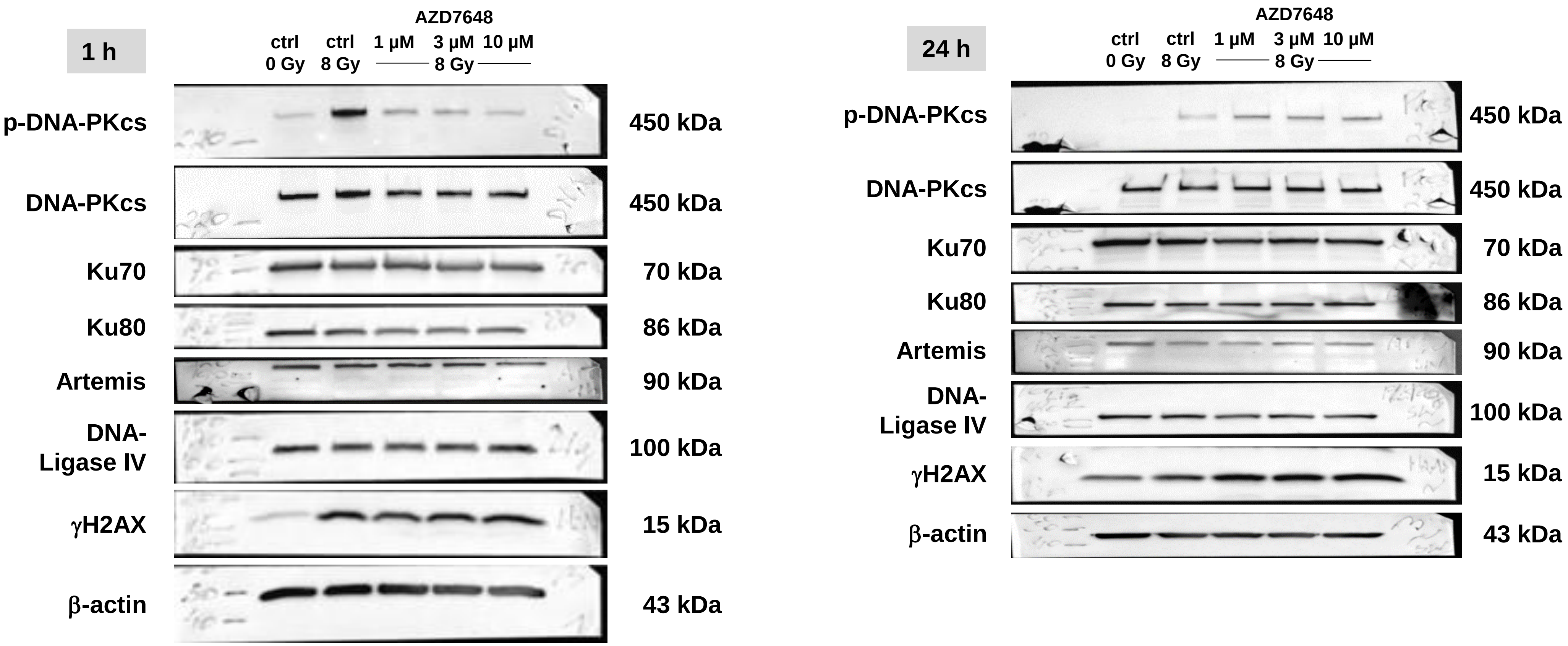

uncropped files_Figure 6B (Cal78; C-ions)

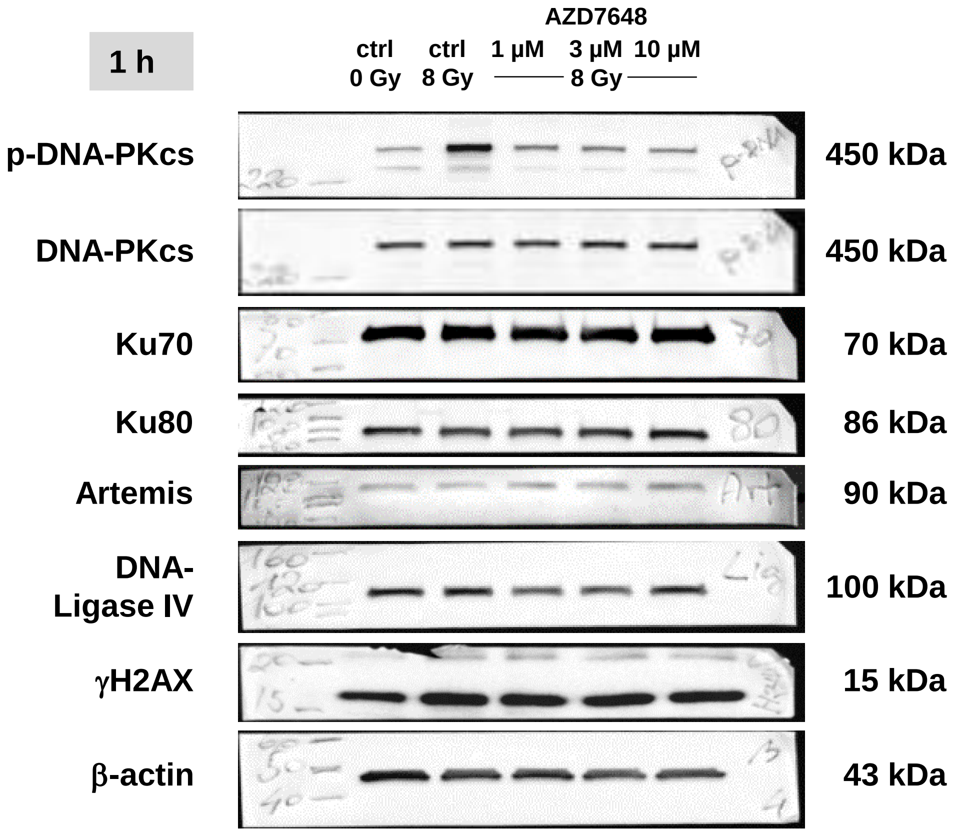

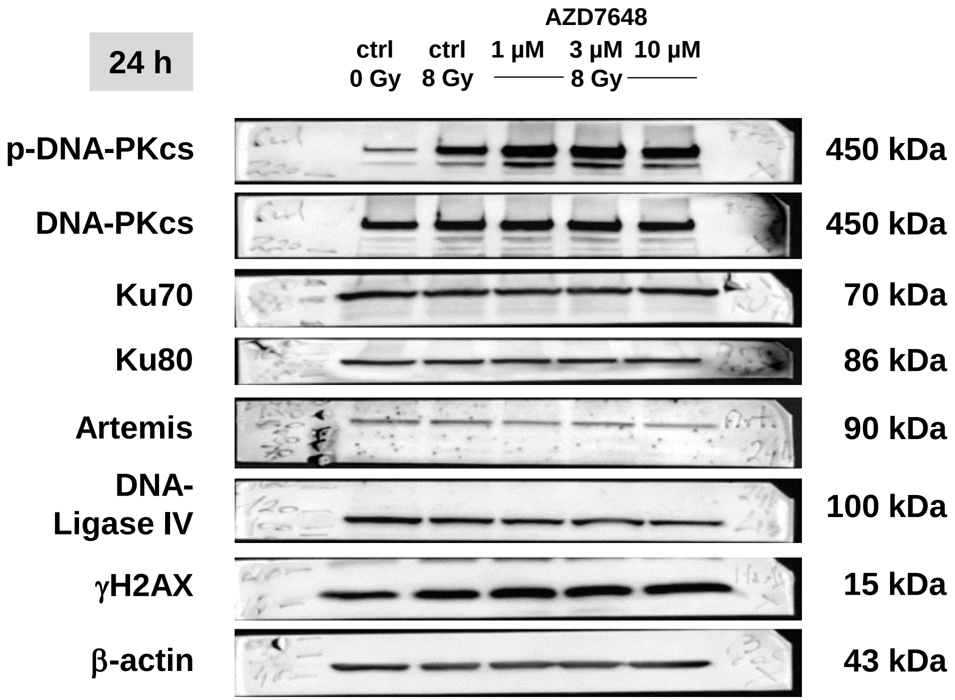

Supplement: Supplementary file 1 [file ijms-25-06179-s001.zip › Supplementary data_uncropped files.pdf]
